# Persistent Unresolved Inflammation in the *Mecp2*-308 Female Mutated Mouse Model of Rett Syndrome

**DOI:** 10.1155/2017/9467819

**Published:** 2017-05-16

**Authors:** Alessio Cortelazzo, Claudio De Felice, Bianca De Filippis, Laura Ricceri, Giovanni Laviola, Silvia Leoncini, Cinzia Signorini, Monica Pescaglini, Roberto Guerranti, Anna Maria Timperio, Lello Zolla, Lucia Ciccoli, Joussef Hayek

**Affiliations:** ^1^Child Neuropsychiatry Unit, University Hospital Azienda Ospedaliera Universitaria Senese (AOUS), Viale M. Bracci 16, 53100 Siena, Italy; ^2^Department of Medical Biotechnologies, University of Siena, Via A. Moro 2, 53100 Siena, Italy; ^3^Clinical Pathology Laboratory Unit, University Hospital AOUS, Viale M. Bracci 16, 53100 Siena, Italy; ^4^Neonatal Intensive Care Unit, University Hospital AOUS, Viale M. Bracci 16, 53100 Siena, Italy; ^5^Centre for Behavioural Sciences and Mental Health, Istituto Superiore di Sanità (ISS), Viale Regina Elena 299, 00161 Rome, Italy; ^6^Department of Molecular and Developmental Medicine, University of Siena, Via A. Moro 6, 53100 Siena, Italy; ^7^Department of Ecological and Biological Sciences, University of Tuscia, Largo dell'Università, snc, 01100 Viterbo, Italy

## Abstract

Rett syndrome (RTT) is a rare neurodevelopmental disorder usually caused by mutations in the X-linked gene *methyl-CpG-binding protein 2* (*MECP2*). Several *Mecp2* mutant mouse lines have been developed recapitulating part of the clinical features. In particular, *Mecp2*-308 female heterozygous mice, bearing a truncating mutation, are a validated model of the disease. While recent data suggest a role for inflammation in RTT, little information on the inflammatory status in murine models of the disease is available. Here, we investigated the inflammatory status by proteomic 2-DE/MALDI-ToF/ToF analyses in symptomatic *Mecp2*-308 female mice. Ten differentially expressed proteins were evidenced in the *Mecp2*-308 mutated plasma proteome. In particular, 5 positive acute-phase response (APR) proteins increased (i.e., kininogen-1, alpha-fetoprotein, mannose-binding protein C, alpha-1-antitrypsin, and alpha-2-macroglobulin), and 3 negative APR reactants were decreased (i.e., serotransferrin, albumin, and apolipoprotein A1). CD5 antigen-like and vitamin D-binding protein, two proteins strictly related to inflammation, were also changed. These results indicate for the first time a persistent unresolved inflammation of unknown origin in the *Mecp2*-308 mouse model.

## 1. Introduction

Rett syndrome (RTT, MIM 312750) is a progressive neurodevelopmental disorder, affecting almost exclusively the female gender. With a frequency of approximately 1 : 10,000 live births, it is a leading cause of severe intellectual disability and autistic features in females [1, 2]. The classical clinical picture of the disease [3] is characterized by a period of 6 to 18 months of apparently normal neurodevelopment followed by an early neurological regression, with a progressive loss of acquired cognitive, social, and motor skills in a typical 4-stage neurological regression pattern [4, 5]. Other known features include stereotypic hand movements, communication dysfunction, seizures, postural hypotonia, tremors, autonomic dysfunction, microcephaly, and growth failure [1]. It has become apparent that there is a spectrum of severity in RTT, as some patients may present with atypical features, sometimes overlapping with autism spectrum disorders [3–5]. RTT is known to be caused in the overwhelming majority of cases by sporadic de novo loss-of-function mutations in the X-linked *methyl-CpG-binding protein 2* (*MECP2*) gene [6] encoding methyl-CpG-binding protein 2 (MeCP2), a nuclear protein that binds to methylated CpGs and regulates gene expression [7, 8]. Different types of mutations within *MECP2* are known to cause RTT, including missense, nonsense, deletions, and insertions [9]. It should be emphasised that most of the proteins identified in mouse serum have orthologs in humans and other mammals [10].

Identification of *Mecp2* as the disease-causing gene led rapidly to the development of mouse models of RTT that recapitulate, to varying degrees, the underlying molecular and genetic defects and symptoms of the human disease [11]. Mouse models range from *Mecp2*-null mutations to specific point mutations mimicking those observed in humans, phenocopying several motor and cognitive features of RTT patients [12–16]. Although mice cannot model all aspects of the human RTT, certainly they recapitulate many features of the disease and are generally accepted as excellent tools to study MeCP2 function [17]. An emerging role for inflammation in human RTT is reported [18–22]. Although *MECP2* is highly expressed in several organs and tissues besides the central nervous system (CNS), including the spleen [16], most of the studies focused on the CNS and little information exists on the inflammatory status in the murine models of the disease, with few exception regarding neuroinflammation processes related to microglia and macrophage function in *Mecp2*-null mice [23]. Furthermore, prior reports provide a link for *MECP2*-RTT to increase erythrocyte sedimentation rate and enhance expression of acute-phase response (APR) proteins and cytokine dysregulation [18–22]. APR is a complex systemic early-defense system activated by trauma, infection, stress, neoplasia, and inflammation [24]. Although nonspecific, it serves as a core of the innate immune response involving physical and molecular barriers and responses that serve to prevent infection, clear potential pathogens, initiate inflammatory processes, and contribute to resolution and the healing process. APR proteins, an integral part of the APR process, have been a focus of many applications in human diagnostic medicine and recently have been identified in common animal species [24]. The number of protein changes was found to be proportional to the severity of the mutation. Our findings revealed for the first time the presence of a subclinical chronic inflammatory status related to the severity carried by the *MECP2* gene mutation. Therefore, inflammation seems to be a previously unrecognized feature in RTT, which could play a role in the evolution of the pathology and its severity. Although the genetic mechanisms of RTT have been explored to an extraordinary extent, to date, the details of the biological mechanisms linking the *MECP2* gene mutation to protein expression as a function of clinical phenotype are yet to be clarified.

2-DE/MALDI-ToF/ToF is extremely helpful in order to understand global protein landscape changes in a given biological fluids and/or tissues for a given condition and is considered to be the “gold standard” technique for identifying and characterizing proteins when fitting a series of different parameters, such as peptide matches, sequence coverage (%), MOWSE score, and pI/Mr (kDa) [25]. In particular, with the single exception of two proteomic studies on mouse models [26, 27], very little information exists on possible *Mecp2* mouse model-related proteome changes. In the present study, a validated mouse model of RTT was used, that is, the *Mecp2*-308 mutated model. This model bears a truncating mutation, leading to the expression of a protein truncated at amino acid 308 [16, 28]. Several lines of evidence suggest that the truncated Mecp2 protein retains at least some of its functions (i.e., its ability to regulate BDNF gene expression, a *Mecp2* target gene) [29], thus contributing to the milder neurobehavioral phenotype of those mice. Based on the previous data demonstrating a clear phenotype in *Mecp2*-308 females [30], the present study was carried out in symptomatic heterozygous female mice, the genetic and hormonal milieus that, from a translational point of view, is closer to the ones of RTT patients; indeed, among RTT patients, *MECP2* mutations are missense and truncated mutations, not deletions [2, 11].

The aim of the present study was to investigate in plasma the occurrence of an inflammatory status in the experimental *Mecp2*-308 mutated Rett female mouse model by using a proteomic 2-DE/MALDI-ToF/ToF approach.

## 2. Materials and Methods

### 2.1. Animals

The experimental subjects were 10–12-month-old *Mecp2*-308 heterozygous (Het) female mice [B6.129S-MeCP2tm1Hzo/J, stock number: 005439] and wild-type littermates. Mice were housed in polycarbonate transparent cages (33 × 13 × 14 cm) with sawdust bedding and kept on a 12 h light-dark schedule (lights off at 8:00). Temperature was maintained at 21 ± 1°C and relative humidity at 60 ± 10%. Animals were provided ad libitum with a complete pellet diet (Altromin, Germany). All procedures were carried out in accordance with the European Communities Council Directive (10/63/EU) and formally approved by the Italian Ministry of Health.

### 2.2. Blood Sampling

Blood samples were centrifuged at 2400*g* for 15 min at 4°C and plasma was collected. All the manipulations were carried out within 2 h after sample collection.

### 2.3. Electrophoretic Separation of Plasma Proteins after Albumin and IgG Depletion

Albumin and IgG were removed using the ProteoPrep Immunoaffinity Albumin & IgG Depletion Kit (Sigma-Aldrich), and two-dimensional gel electrophoresis (2-DE) was performed according to Görg et al. [31]. Samples containing 60 *μ*g of protein as determined by Bradford [32] were denatured with 10 ml of a solution containing 10% of SDS and 2.3% of dithiothreitol (DTT). Afterwards, samples were combined with 350 ml of solubilizing buffer containing 8 M urea, 2% of 3-[(3-cholamidopropyl)-dimethylammonium]-1-propane sulfonate, 0.3% DTT, and 2% of immobilized pH gradient (IPG) buffer, loaded into 18 cm IPG strips (pH 3–10) nonlinear on an Ettan IPGphor Apparatus system (GE Healthcare), and rehydrated for 7 h. IEF was carried out for a total of 32 kVh. After focusing, the strips were equilibrated with the buffer containing 50 mM Tris-HCl (pH 8.8), 6 M urea, 2% *w*/*v* SDS, 30% *v*/*v* glycerol, and 1% *w*/*v* DTT for 15 min. Subsequently, strips were equilibrated again with the same equilibration buffer described above, except that it contained 4% *w*/*v* iodoacetamide instead of DTT and a trace of bromophenol blue. IPG strips and a molecular weight standard were embedded at the top of a 1.5 mm thick vertical polyacrylamide gradient gel (8–16% T) using 0.5% *w*/*v* agarose and run at a constant current of 40 mA/gel at 20°C. The second dimension was performed on an Ettan Daltsix Electrophoresis system (GE Healthcare). Each sample was carried out in triplicate under the same conditions.

### 2.4. Protein Identification

After mass spectrometry-compatible silver staining, the preparative gel was matched to the master gel in the analytical gel match set [33]. A spot-picking list was generated and exported to Ettan Spot Picker (GE Healthcare). The spots were excised and delivered into 96-well microplates where they were destained and dehydrated with acetonitrile (ACN) for subsequent rehydration with trypsin solution. Tryptic digestion was carried out overnight at 37°C. Each protein spot digest (0.75 ml) was spotted into the MALDI instrument target and allowed to dry. Then, 0.75 ml of the instrument matrix solution (saturated solution of *α*-cyano-4-hydroxycinnamic acid in 50% ACN and 0.5% *v*/*v* trifluoroacetic acid) was applied to dried samples which were dried again. Mass spectra were obtained using an ultrafleXtreme MALDI-ToF/ToF (Bruker Corporation, Billerica, MA, USA), as previously described [34]. After tryptic peptide mass acquisition, mass fingerprint searching was carried out in Swiss-Prot/TREMBL and NCBInr databases using MASCOT (Matrix Science, London, UK, http://www.matrixscience.com). A mass tolerance of 100 ppm was allowed and only one missed cleavage site was accepted. Alkylation of cysteine by carbamidomethylation was assumed as a fixed modification, whereas oxidation of methionine was considered a possible modification. Criteria used to accept identifications included the extent of sequence coverage, number of matched peptides, and probabilistic scores. Tryptic digests that did not produce MALDI-TOF unambiguous identifications were subjected to ESI-TRAP MS/MS peptide sequencing on a nanospray/LCQ Deca ion trap mass spectrometer (Thermo Finnigan, San Jose, CA, USA).

### 2.5. Image and Data Analysis

Gel imaging was performed by using ImageMaster 2D Platinum v7.0 software (GE Healthcare). A reference gel for each group was defined for the comparative analyses. The background was subtracted from all gels using the average on-boundary method. Spot volume was expressed as a ratio of the percentage volume (%V) detected from the entire gel to minimize differences between samples (i.e., normalization). Unmatched spots or spots with significantly different %V were considered differently expressed. Data were expressed as mean ± SD/SEM or medians [95% CI for median] as appropriate. Statistical analysis of protein variations was performed using multiple *t*-test with a false discovery rate (q) of 0.05. Differences between groups were tested using Kruskal-Wallis test or one-way ANOVA, with Dunn's or Holm-Sidak's multiple-comparisons tests for post hoc analyses. A two-tailed *P* value of less than 0.05 was considered statistically significant. The statistical software GraphPad Prism v6.01 (GraphPad Software Inc., La Jolla, CA, USA) and MedCalc v12.1.4 software package (MedCalc Software, Mariakerke, Belgium) were used.

## 3. Results

### 3.1. Protein Expression Profile Differences between Mecp2-308 and Wild-Type Mouse Models

**Figure 1:**
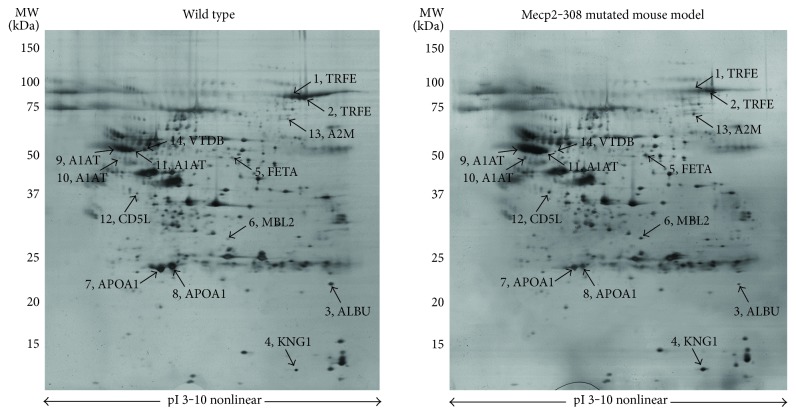
Silver-stained 2-DE of proteins from wild-type and *Mecp2*-308 mutated mouse models. A total of 60 μg of protein from albumin-depleted plasma samples was subjected to the first dimension electrophoresis on IPG strips, with nonlinear pH ranging from 3 to 10 (pI, isoelectric point), followed by SDS-polyacrylamide gradient gel (8–16% T) electrophoresis. Molecular mass (MW, kDa) and pI markers are indicated along the gels. Numbers followed by short names denote the mass spectrometry-identified protein spots listed in Tables 1 and 2.

Expression changes for a total of 10 differentially expressed proteins were identified in the *Mecp2*-308 mutated model, relative to wild type, and are shown in 2-DE maps (Figure 1). The proteins were subsequently identified by mass spectrometry. Protein identification as well as peptide matches, sequence coverage, and the probabilistic score was obtained using the MASCOT software (Table 1). A total of 14 spots were identified as follows: serotransferrin (TRFE spots 1 and 2), albumin (ALBU, spot 3 as a fragment), kininogen-1 (KNG1, spot 4), alpha-fetoprotein (FETA, spot 5), mannose-binding protein C (MBL2, spot 6), apolipoprotein A1 (APOA1, spots 7 and 8), alpha-1-antitrypsin (A1AT, spots 9, 10, and 11), CD5 antigen-like (CD5L, spot 12), alpha-2-macroglobulin (A2M, spot 13), and vitamin D-binding protein (VTDB, spot 14). A two-way analysis of variance showed significant between-subjects effects (proteins, F: 2157.84, DF: 13, *P* < 0.001; mouse category (mutated versus wild type), F: 994.35, DF: 1, *P* < 0.001; and proteins^∗^mouse category, F: 866.18, DF: 13, *P* < 0.001).

**Figure 2:**
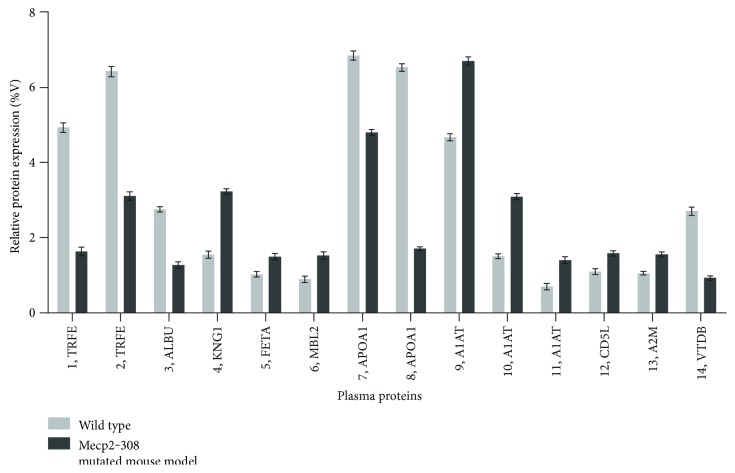
Expression analysis of plasma proteins from wild-type and *Mecp2*-308 female mutated mouse models. Expression changes of proteins are reported as mean ± SEM and are statistically significant. Numbers followed by short names refer to proteins reported in Figure 1.

KNG1, FETA, MBL2, A1AT, and A2M were overexpressed in the examined *Mecp2*-308 mutated mouse model (Figures 1 and 2, Supplementary material, Supplementary Table 1 available online at https://doi.org/10.1155/2017/9467819). In particular, KNG1 and A2M correspond to fragments (KNG1, experimental MW: 12.6 kDa; A2M, experimental MW: 64.2 kDa), both considered products of the entire proteins. Interestingly, all the identified A1AT spots were significantly overexpressed (*P* < 0.001) in the *Mecp2*-308 mutated mouse model.

TRFE, ALBU, and APOA1 were found to be underexpressed in the mutant mouse model. In particular, ALBU was identified as a fragment (experimental MW: 22.6 kDa), a product of the entire protein (theoretically mapped at MW 70.7 kDa). Of note, the CD5L spot 12 was overexpressed (*P* < 0.05), while the VTDB spot 14 was significantly underexpressed (*P* < 0.001) in the plasma pattern of the *Mecp2*-308 mutated murine model.

### 3.2. Biological Functions and APR Role of Differentially Expressed Plasma Proteins in the Mecp2-308 Female Mouse Model

Functions of differentially expressed proteins were evaluated in greater detail specifically for mice with the use of ExPASy, a bioinformatics resource portal operated by the Swiss Institute of Bioinformatics (SIB) (https://www.expasy.org/). The majority of the proteins can be categorized as either positive APR reactants (i.e., KNG1, FETA, MBL2, A1AT, and A2M) or negative APR proteins (i.e., TRFE, ALBU, and APOA1). KNG1, A1AT, and A2M, three protease inhibitors, are strictly related to inflammatory processes and coagulation mechanisms [35–37]. TRFE, FETA, MBL2, ALBU, APOA1, and VTDB are known to be mainly binding and transport proteins for bioactive molecules and/or inflammatory mediators [38–43]. CD5L is a key regulator of inflammatory response mechanisms and is a macrophage-secreted glycoprotein mainly expressed in lymphoid and inflamed tissues, regulating the tissue macrophage homeostasis [44, 45]. A summary of the biological functions and APR role of the differentially expressed plasma proteins in the *Mecp2*-308 female model is shown in Table 2.

## 4. Discussion

Our findings indicate for the first time the presence of a persistent unresolved inflammatory status in the *Mecp2*-308 female mutated mouse model of RTT. The inflammatory protein pattern was found to be mainly characterized by an attenuated APR, with evident similarities to that of the human disease. The attenuated APR pattern, observed in the *Mecp2*-308 mutated murine model, shares some relevant features to the protein changes in the plasma protein pattern from RTT girls [18, 46–48]. In particular, plasma from *Mecp2*-308 mutant mouse shows increased levels of the positive APR reactant A1AT, a multifunctional protein involved in anti-inflammatory and tissue protective properties. More broadly, A1AT plays an important role in modulating immunity, inflammation, apoptosis, and possibly cellular senescence programs [36].

Interestingly, 4 well-known positive APR reactants (i.e., KNG1, FETA, MBL2, and A2M), not previously evidenced in the plasma from RTT patients, specifically characterize the *Mecp2*-308 inflammatory response. Apart from their typical APR function, these proteins are also involved in the coagulation process (i.e., KNG1 and A2M) [35, 37], fatty acid and metal binding (i.e., FETA) [39], and innate immune defense and inflammatory modulation (i.e., MBL2) [40]. In particular, KNG1 and A2M are two well-known protease inhibitors. KNG1 fragments possess biological activity with moderate to strong correlation with specific diseases (i.e., early progressive renal function decline with type 1 diabetes) [35], while those of murine A2M increase during inflammatory responses and tumor growth [37]. Likewise, FETA shows close connections with the modulation of the proinflammatory response of human keratinocytes [39]. Of note, MBL2 comprises a cysteine-rich domain at the *N*-terminus followed by a collagen-like domain, a neck region, and a carbohydrate recognition domain at the *C*-terminus [40]. This domain recognizes and binds to chemical patterns, which include D-mannose, L-fucose, and GlcNAc, carbohydrate residues found on the surface of many pathogens. Furthermore, its structural features suggest a possible role in opsonisation, activating a complement through the lectin complement pathway [40].

A key novel finding for the *Mecp2*-308 plasma protein pattern is represented by the increase in CD5L coupled with the decrease in VTDB. These two proteins, although not strictly APR reactants, are closely related to inflammatory processes [43–45].

CD5L expression appears to be increased in activated macrophages thus regulating inflammatory response processes [49, 44, 45]. In particular, CD5L is known to prevent Toll-like receptor-induced TNF-*α* and IL-1*β* secretion, with a concomitant increase in IL-10 levels, thereby downregulating the tissue macrophage inflammatory reaction [45]. An increased CD5L expression was recently evidenced in plasma from RTT girls in the presence of an inflammatory APR protein response [48]. CD5L induction or upregulation were also reported in response to inflammatory stimuli depending on the variety of physiological situations [49, 50]. However, the basic mechanism of the CD5L expression induction could depend on a microenvironment (i.e., cell-cell interaction between a specific type of cells and macrophages) [49]. Therefore, also cytokines produced by lymphocytes could contribute to the upregulation of CD5L expression [49]. Interestingly, circulating levels of several cytokines including macrophage-related cytokines such as TNF-*α*, IL-6, IL-12p70, IL-10, TGF-*β*1, IL-8, and RANTES were found to be abnormal in RTT girls [22]. In particular, a strongly increased release of IL-10 was evidenced [22]. The evidenced positive correlation between CD5L and IL-10 suggests a general hyperactivity of the macrophage component of RTT [48]. Several of the observed cytokine pattern changes in *MECP2*-RTT girls [22] appear to reflect a likely macrophage dysregulation/dysfunction as previously suggested for *Mecp2*-null mice [19], demonstrating that MeCP2 acts as a regulator of the response to inflammatory stimuli in the microglia and macrophages. Further study will precisely clarify the elements required for CD5L expression induction in RTT. Of note, VTDB levels are reported to be decreased in acute inflammatory conditions [43]. Moreover, VTDB impacts on 25-hydroxyvitamin D levels under different physiologic and pathologic conditions [43]. Furthermore, three negative reactants (i.e., TRFE, ALBU, and APOA1) significantly changed in their expression, in line with our prior study [18]. Alterations in the TRFE levels may lead to abnormal iron metabolism in RTT [38]. Expression of TRFE, previously reported to be involved in autism pathophysiology, decreases during inflammation [38]. Thus, the observed underexpression of TRFE in RTT, as well as in the *Mecp2*-308 mutant murine model, would suggest once again that an inflammatory process could play a key role in the pathogenesis of the disease. ALBU is involved in the regulation of the osmotic blood pressure, ions, hormones, and fatty acid binding [41]. Finally, APOA1, a principal component of high-density lipoproteins, shows an impressive portfolio of anti-inflammatory mechanisms at the interface of vascular inflammation and inflamed tissue [42]. Its list of properties includes inhibition of endothelial cell adhesion molecule expression, myeloid lineage cell proliferation, and expression of chemokine receptors and cytokines in synovial lining cells, and neutrophils ingress into inflamed tissues [42].

Very little is known on inflammation and the APR process in *Mep2*-308 mutated mouse model, as well as in other experimental mice. In *Mecp2*-308 male mouse brains, an abnormal profile of cytokines has been observed (i.e., IL-6 was dramatically overexpressed, while TNF-*α* was downregulated), thus suggesting that the morphological deficits of astrocytes are accompanied by aberrant functionality. On the other hand, no data are available on plasma cytokine levels in this *Mecp2* mouse line. Recently, high levels of TNF-*α*, IL-6, and IL-3 have been reported in an in vitro human peripheral blood mononuclear cell (PBMC) experimental setting in which the *MECP2* gene was silenced [21].

Clinical studies support the presence of differences in the clinical manifestation of the syndrome in RTT patients carrying different mutations in the *MECP2* gene. From a translational point of view, this is consistent with the profound differences in the symptom gravity and in the age of their onset exhibited by the various mouse models developed. Indeed, different RTT models are characterized by different genetic mutations and by consequent different degrees of protein integrity [17]. We have chosen to study the *Mecp2*-308 RTT mouse model since among RTT patients, *MECP2* mutations are missense and truncated mutations, so this model more closely resembles the clinical phenotype shown by the majority of the patients compared to null models. In particular, the *Mecp2*-308 model is characterized by a delayed onset of symptoms and a prolonged life-span [16, 28] in comparison with models in which the gene is deleted. Importantly, the behavioral phenotype of *Mecp2*-308 recapitulates most of the clinical phenotype shown by RTT patients carrying C-terminal deletions of the *MECP2* gene [51].

Reduction in several plasma proteins, potentially affecting cholesterol transport and inhibiting oxidation phenomena, is known to occur during inflammation. These proteins include cholesterol ester transfer protein, hepatic lipase, and apolipoproteins. It is thought that reduction in these proteins, associated with an increase in positive APR proteins, may change the high-density lipoprotein from anti-inflammatory into proinflammatory particles [52].

A recent report, analyzing the response of astrocytes during activation by proinflammatory stimulation, proposes that the protective phenotype against iron-mediated oxidative stress in cell death involves a complex change in the expression and activity of several genes involved in the control of the cellular redox state [53]. Therefore, it is plausible that abnormal redox status and unrecognized proinflammatory stimuli would converge in RTT patients to functionally damage the astrocytes within the CNS. There is a mounting evidence for a persistent redox imbalance in RTT [54–56]. This abnormal redox homeostasis could be related to the wider context of an unresolved inflammatory process whose fine mechanisms remain to be elucidated.

Inflammation plays an important role in the pathophysiology of several common diseases, such as Alzheimer's and atherosclerosis. Thus, the cellular mechanisms by which resolution occurs and the key biochemical pathways associated with the return to homeostasis/catabasis (return from disease) clearly open many new avenues for potential therapeutic interventions in a wide range of diseases associated with unresolved inflammation [57].

RTT appears to be more complex than previously thought. A gene sequence analysis has indicated several hundreds of gene mutations associated with the *MECP2* gene mutation and, therefore, to be considered potential disease modifiers [58].

*MECP2* levels start to increase postnatally with the protein being abundant in the mature nervous system [59]. MeCP2 protein expression is not uniform in different neuronal cell populations and parts of the brain [60] and is known to change with age [61]. MeCP2 is a multifunctional protein influencing gene expression and metabolism on many levels [62]. MeCP2 is currently considered a global, genome-wide transcriptional regulator (i.e., mainly repressor) whose function is well correlated with DNA methylation density for a large proportion of unusually long brain-specific genes [62]. Likely, a loss of MeCP2 function causes a transcriptional imbalance leading to a suboptimal brain homeostasis [63]. A clear cut proof-of-concept demonstration for the effects of *MeCP2* gene mutations on the homeostasis of the brain is the absence of major phenotypes associated with Rett-like mice when levels of the central nervous system MeCP2 are normal [64]. *MECP2* dampens neuronal transcription globally, thus allowing activity-related responses essential for the learning process and specific synapsis formation [65, 66].

Although the genetic mechanisms of RTT have been explored to an extraordinary extent, to date the details of the biological mechanisms linking the *MECP2* gene mutation to protein expression as a function of clinical phenotype have yet to be clarified. The findings of the present study add a further phenotypical feature, thus expanding our knowledge on the molecular cascade of events that lead to the disease phenotype.

## 5. Conclusion

Our results indicate for the first time the occurrence of a persistent unresolved inflammation of unknown origin in the *Mecp2*-308 female mouse model of RTT, which appears to be helpful to elucidate in greater detail the importance of the inflammatory status, disentangle the role of inflammation in RTT on the phenotype, and contribute in developing inflammation-targeted therapeutic strategies.

## Supplementary Material

Supplementary Table 1. Details for differentially expressed plasma proteins in the female Mecp2-308 and wild type mouse model; Gel Analysis Table

## Figures and Tables

**Table 1:** Summary of proteins identified in the *Mecp2*-308 plasma proteome.

Spot	SwissProt code	Protein name	Short name	Theoretical pI/Mr (kDa)	Peptide matches	Sequence coverage (%)	MOWSE score
1	Q921I1	Serotransferrin	TRFE	6.81/79.2	5/8	8	154
2	Q921I1	Serotransferrin	TRFE	6.81/79.2	9/20	16	272
3	P07724	Albumin	ALBU	5.75/70.7	17/38	26	483
4	O08677	Kininogen-1	KNG1	6.05/74.1	6/9	9	219
5	P02772	Alpha-fetoprotein	FETA	5.47/48.7	5/8	11	148
6	P41317	Mannose-binding protein C	MBL2	4.96/26.3	3/6	14	93
7	Q00623	Apolipoprotein A1	APOA1	5.64/30.5	11/35	31	280
8	Q00623	Apolipoprotein A1	APOA1	5.64/30.5	10/35	36	239
9	P07758	Alpha-1-antitrypsin	A1AT	5.33/46.0	4/10	9	81
10	P07758	Alpha-1-antitrypsin	A1AT	5.33/46.0	15/50	30	804
11	P07758	Alpha-1-antitrypsin	A1AT	5.33/46.0	6/11	19	143
12	Q9QWK4	CD5 antigen-like	CD5L	5.01/40.3	4/7	13	133
13	Q61838	Alpha-2-macroglobulin	A2M	6.27/167.0	11/25	8	370
14	P21614	Vitamin D-binding protein	VTDB	5.26/54.6	14/25	7	86

Spot numbers match those reported in the representative 2-DE images in Figure 1.

**Table 2:** Biological functions and APR role of the differentially expressed plasma proteins in the *Mecp2*-308 female mouse model.

Protein name	Short name	Biological functions (source: ExPASy)	APR proteins
Serotransferrin	TRFE	Iron binding and transport	(−)
Albumin	ALBU	Transport and regulation of colloidal osmotic pressure	(−)
Kininogen-1	KNG1	Mediator of inflammation and coagulation and protease inhibitor	(+)
Alpha-fetoprotein	FETA	Inflammation and fatty acid and metal binding	(+)
Mannose-binding protein C	MBL2	Innate immune defense and inflammation modulation	(+)
Apolipoprotein A1	APOA1	Lipid transport and metabolism	(−)
Alpha-1-antitrypsin	A1AT	Inflammation, coagulation, and protease inhibition	(+)
CD5 antigen-like	CD5L	Regulation of mechanisms in inflammatory responses	NA
Alpha-2-macroglobulin	A2M	Inflammation, coagulation, and protease inhibition	(+)
Vitamin D-binding protein	VTDB	Vitamin D sterol carrier	NA

APR: acute phase response; (+): positive APR proteins; (−): negative APR proteins; NA: not applicable.

## Abbreviations

A1AT: Alpha-1-antitrypsin
A2M: Alpha-2-macroglobulin
ALBU: Albumin
APOA1: Apolipoprotein A1
CD5L: CD5 antigen-like
FETA: Alpha-fetoprotein
KNG1: Kininogen-1
MBL2: Mannose-binding protein C
TRFE: Serotransferrin
VTDB: Vitamin D-binding protein.
