# Supplementary material for: Persistent Unresolved Inflammation in the Mecp2-308 Female Mutated Mouse Model of Rett Syndrome
Source: Mediators Inflamm. 2017 May 16;2017:9467819. doi: 10.1155/2017/9467819 (PMC5448068; doi:10.1155/2017/9467819)
Supplement: Supplementary file 1 — Supplementary Table 1. Details for differentially expressed plasma proteins in the female Mecp2-308 and wild type mouse model; Gel Analysis Table [file 9467819.f1.doc]

**Supplementary Table 1.** Details for differentially expressed plasma proteins in the female *Mecp2*-308 and wild type mouse model

| **Spot** | **Protein name** | **Short name** | **Wild type** | ***Mecp2*-308** | ***P-*value** |
| --- | --- | --- | --- | --- | --- |
| 1 | Serotransferrin | TRFE | 4.92±0.12 | 1.63±0.11 | 6.26509E-05 |
| 2 | Serotransferrin | TRFE | 6.42±0.13 | 3.11±0.10 | 0.00073 |
| 3 | Albumin | ALBU | 2.75±0.07 | 1.28±0.08 | 0.00360 |
| 4 | Kininogen-1 | KNG1 | 1.55±0.09 | 3.23±0.06 | 0.00245 |
| 5 | Alpha-fetoprotein | FETA | 1.03±0.07 | 1.49±0.08 | 0.00986 |
| 6 | Mannose-binding protein C | MBL2 | 0.90±0.08 | 1.53±0.09 | 0.01180 |
| 7 | Apolipoprotein A1 | APOA1 | 6.85±0.12 | 4.80±0.07 | 0.00014 |
| 8 | Apolipoprotein A1 | APOA1 | 6.53±0.10 | 1.71±0.04 | 0.00022 |
| 9 | Alpha-1-antitrypsin | A1AT | 4.67±0.09 | 6.70±0.11 | 0.00202 |
| 10 | Alpha-1-antitrypsin | A1AT | 1.51±0.06 | 3.09±0.08 | 0.00040 |
| 11 | Alpha-1-antitrypsin | A1AT | 0.70±0.08 | 1.41±0.09 | 8.40658E-05 |
| 12 | CD5 antigen-like | CD5L | 1.10±0.07 | 1.59±0.06 | 0.01934 |
| 13 | Alpha-2-macroglobulin | A2M | 1.06±0.04 | 1.56±0.06 | 0.00091 |
| 14 | Vitamin D-binding protein | VTDB | 2.71±0.10 | 0.93±0.05 | 0.00267 |

Spot numbers match those reported in the representative 2-DE images in Figure 1. Levels of proteins expression are reported as means ± SD.
